# A cohort study of both human menopausal gonadotropin (HMG) and recombinant luteinizing hormone addition at early follicular stage in in vitro fertilization outcome: A STROBE-compliant study

**DOI:** 10.1097/MD.0000000000015512

**Published:** 2019-05-13

**Authors:** Xi Xia, Yu Shi, Lan Geng, Dan Liu, Zhenhui Hou, Hongbo Lin, Rong Li, Haiyan Wang, Liyuan Tao, Fanhua Meng, Jian Da, Yun Chen, Jie Qiao, Weiping Qian, Hongzhen Li

**Affiliations:** a Center for Reproductive Medicine, Department of Obstetrics and Gynecology, Peking University Shenzhen Hospital, No. 1120 Lotus Road, FuTian District, Shenzhen, Guangdong; b Center for Reproductive Medicine, Department of Obstetrics and Gynecology, Peking University Third Hospital, No. 49 North HuaYuan Road, HaiDian District, Beijing, China.

**Keywords:** GnRH agonist, HMG, IVF, recombinant LH (rLH)

## Abstract

At present, the precise role of human menopausal gonadotropin (HMG) and recombinant luteinizing hormone (rLH) supplementation at an early time of follicular phase on in vitro fertilization (IVF)/intra cytoplasmatic sperm injection (ICSI) outcomes remains uncertain.

Here infertile women of normal ovarian function undergoing their first cycle of IVF/ICSI were studied and were randomly allocated into 3 groups. Group 1, ovarian stimulation with 150IU recombinant follicle-stimulating hormone (FSH) alone. Group 2, patients received 75IU rFSH and 75IU HMG. Group 3 patients were given 150IU rFSH and 75IU rLH.

There were no significant differences in the clinical characteristics, ovarian response, the biochemical, clinical and ongoing pregnancy rates among the 3 groups. No significant differences were found in biochemical, clinical and ongoing pregnancy rates between the patients whose LH levels were lower than 0.75 mIU/ml and those above this threshold in group 1. Furthermore, there were also no significant differences in biochemical, clinical and ongoing pregnancy rates among the 3 group patients whose LH level lower than 0.75 mIU/ml.

The results showed that either the addition of HMG or rLH supplementation at an early time of follicular phase produce no significant benefit on IVF outcome in patients with normal ovarian function.

## 1 Introduction

It is well known that not only follicle-stimulating hormone (FSH) but also luteinizing hormone (LH) plays a vital role in the regulation of follicular growth and maturation.^[1–3]^ LH induces theca cells of developing follicles to produce androgens and polypeptide growth factors which enhance follicular response to FSH during follicular recruitment and selection.^[4,5]^ Besides, LH could also stimulate the growth of large antral follicles.^[6,7]^

However, LH supplementation in controlled ovarian stimulation (COS) remains controversial in patients who undergo in vitro fertilization (IVF) ^[8]^. Some studies indicated that normogonadotropic women treated with Gonadotrophine-releasing hormone agonist (GnRHa) might encounter such a profound suppression of LH levels which could lead to a negative effect on IVF outcome. ^[9,10]^ Recently, Anthony reported that the addition of urinary LH, that was low dose human chorionic gonadotropin (hCG), improved implantation and live-birth rates in patients with low LH levels.^[11]^ Meldrum also demonstrated that low LH levels were associated with high rates of early pregnancy loss. ^[12]^ On the contrary, other investigators suggested that the endogenous LH level after down-regulation with GnRHa was sufficient to sustain the development of follicles in normogonadotropic women and no exogenous LH addition was required. The results showed that there were no significant differences in clinical outcomes among different groups of patients with different LH levels after suppression. ^[13]^ Two extensive meta-analysis showed that the supplementation of rLH to general patients was not proved to improve IVF outcomes.^[14,15]^ At present, there are mainly 2 different forms of LH used in clinical medicine including urinary LH that was HMG and recombinant LH. The above contradictive results might be related to the doses and forms of LH added and the timing of LH administration in the different studies.

With the availability of recombinant LH, it is now possible to investigate the precise role of LH supplementation in IVF outcomes and to compare the effect of different forms of LH including rLH and HMG on IVF cycle outcomes in a GnRH agonist protocol among normogonadotropic women.

## 2 Methods

### 2.1 Patients

This retrospective study was reviewed and approved by the ethic committee of the Reproductive Center of Peking University Third Hospital. Women with normal ovarian function (FSH on cycle day 2 was less than 10 IU/L, total number of antral follicles >5 in both ovaries) and undergoing their first IVF/ICSI cycle between Jan 2013 and May 2014 at our center were included. Patients were also excluded if they had any endocrinopathological diseases including Cushing's syndrome, hyperprolactinaemia, adrenal hyperplasia, acromegaly, hypothalamic amenorrhea, hypothyroidism and diabetes mellitus type I.

### 2.2 Ovarian stimulation protocol

For all these women, luteal phase long protocol with GnRH agonist was used and the initial dose of gonadotrophins for ovarian stimulation was 150 IU. Pituitary down-regulation was achieved by administration of 1.8 mg triptorelin (Decapeptyl 3.75; Ipsen Pharma Biotech) on the 21st day of the previous menstrual cycle. Gonadotrophins were started 14 days later when serum estradiol (E2) level was ≤180 pmol/L and no follicle was ≥10 mm in diameter by vaginal ultrasound scan. According to different types of gonadotrophins used, these patients were divided into 3 groups. In Group 1, rFSH (Gonal F; Merck Serono) was started at a dose of 150 IU per day in 277 patients. In Group 2, 161 patients were given 75 IU rFSH combined with 75 IU domestic urinary human menopausal gonadotropin (hMG; Lizhu Pharma) which contains 75 IU urinary FSH and 75 IU urinary LH daily from the beginning of ovarian stimulation. In group 3, 150 IU rFSH combined with 75 IU rLH (Luveris; Merck Serono) were used from the beginning of stimulation in 137 patients. Since this was a retrospective study and the COS regimens in the above 3 different groups were routinely performed in the daily clinical use, the informed consent was not given to the patients. It was up to the doctors to decide the types of gonadotropins being used according to their preference. The serum level of LH after pituitary down-regulation was not as a reference for the choice of gonadotropins at our center. From day 6 of ovarian stimulation, sequential transvaginal ultrasound scan was performed to monitor ovarian response to stimulation. Serum levels of LH, E2, and progesterone (P) were measured on day 6, day of hCG administration or any day needed. The dosage of gonadotropins was adjusted according to ovarian response. When there were at least 2 follicles ≥18 mm in diameter, 250 μg recombinant hCG (rhCG, Ovidrel, Merck-Serono SA) was administered. Oocyte retrieval was performed 36–37 hours after hCG administration. Oocytes and embryos were evaluated according to published criteria. ^[16]^ No more than 2 embryos were transferred either on 3 or 5 days after oocyte retrieval. From the day of oocyte retrieval, luteal phase support was started with 60 mg P intramuscularly or 90 mg P gel (Crinone, Merck, Serono) vaginally once a day.

### 2.3 Pregnancy outcome definition

Serum hCG was measured 14 days after embryo transfer. Biochemical pregnancy was defined when serum hCG level was ≥30 IU/L. Clinical pregnancy was diagnosed when at least one gestational sac was found by ultrasonography on the 28th day after embryo transfer. Ongoing pregnancy was diagnosed when a pregnancy was beyond 12 weeks of gestational age. A miscarriage was defined by the spontaneous loss of a clinical pregnancy before 12 weeks of gestational age.

### 2.4 Hormonal measurements

Blood samples were collected to measure the E2, FSH, LH, P, and testosterone on cycle day 3, stimulation period, day of hCG administration and day of embryo transfer. All serum samples were assayed by professional staffs in the endocrine laboratory of the Reproductive Center of Peking University Third Hospital. The concentrations of hormones were determined by IMMULITE 2000 chemiluminescence immune detection system (Siemens, Erlangen, Germany). LH and FSH were performed using double antibody sandwich immunoassay, with lower detection limit of 0.05 IU/L and 0.1 IU/L for LH and FSH, respectively. E2, P, and testosterone were measured by competitive immunoassay, with the lowest detection limit of 73 pmol/L for E2 and 0.6 nmol/L for P and 0.69 nmol/L for testosterone.

### 2.5 Statistical analysis

Statistical analyses were carried out using the SPSS 18.0 program software (SPSS, Inc., Chicago, IL). Continuous data which follow the normal distribution in the general population were presented as mean ± standard deviation, and were compared between groups with the one-way ANOVA or the independent *t* test as appropriate. Continuous data which do not follow the normal distribution in the general population were presented as median (interquartile range) and comparison between groups was carried out using the Mann–Whitney *U* test. Categorical data were presented as counts (percentages), comparison between groups was performed using the Pearson *χ*2 test or Fisher exact test as appropriate. For all analysis, 2-sided *P* < .05 indicated a statistically significant.

## 3 Results

These patients were followed up between Jan 2013 and January 2015 at our center. The overall mean age of the women was 30.0 ± 3.4 years old (range 21–40 years). The indications for IVF/ICSI included tubal factor (55.5%), male factor (35.3%), unexplained infertility (7.1%), endometriosis and polycystic ovarian syndrome (PCOS) (2.1%). A total of 575 patients were analyzed. There were 277, 161, and 137 patients in Group 1, 2, and 3, respectively. The baseline characteristics of the patients in each group were comparable and there was no significant difference in women's age, body mass index (BMI), duration of infertility and causes of infertility. These were summarized in Table 1.

**Table 1:**
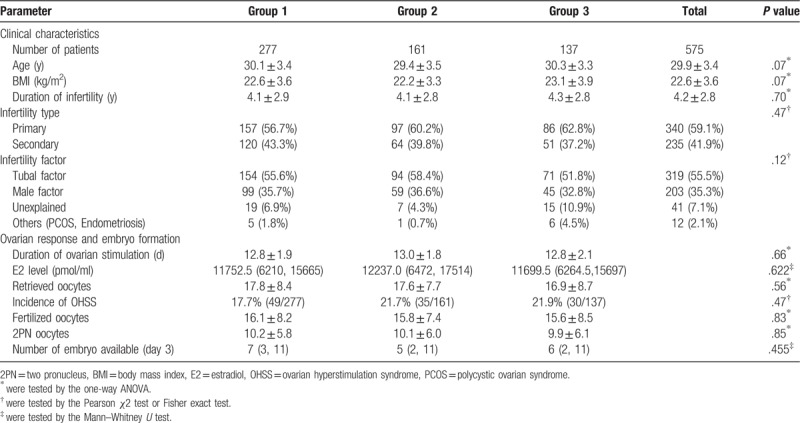
Patients’ clinical characteristics, ovarian response and embryo formation in different groups.

Table 1 also showed the ovarian response and embryo formation in the 3 groups. There was no significant difference in ovarian response regarding to the days of stimulation, the number of oocytes retrieved and E2 level on the day of hCG administration. The incidence of ovarian hyperstimulation syndrome (OHSS) in the 3 groups were 17.7%, 21.7%, and 21.9%, respectively, which showed no significant difference. The number of fertilized oocytes were 16.1, 15.8, and 15.6 in the 3 groups which also showed no significant difference. There was also no significant difference among the 3 groups in the number of 2 pronucleus (2PN) formation and the embryos available on day 3.

The embryo transfer rates in 3 groups were 78% (216/277), 77% (124/161), and 73% (100/137), respectively. The biochemical pregnancy rates were 63.0%, 65.3%, and 64.0% in Group 1, 2, and 3, respectively, which were similar among 3 groups. Although the clinical pregnancy rate in Group 3 (63.0%) was a little higher than those in Group 1 (59.3%) and Group 2 (58.9%), the difference was not statistically significant. The ongoing pregnancy rates were 52.8%, 53.2%, and 55.0% in the 3 groups, respectively, which also showed no significant differences (Table 2).

**Table 2:**
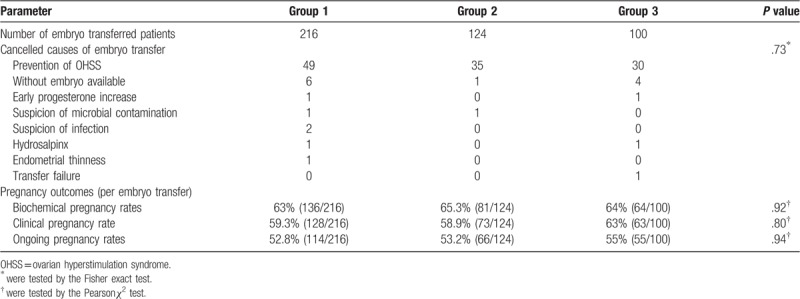
Pregnancy outcome in the 3 groups.

Table 3 showed the IVF outcomes within Group 1 (FSH-only group) according to endogenous LH levels after down-regulation (lower than 0.75 IU/L or above). Patients whose LH levels were lower than 0.75 IU/L had lower biochemical (59.6% vs 63.9%), clinical (55.3% vs 60.4%) and ongoing (42.6% vs 55.6%) pregnancy rates compared to those whose LH levels were higher than 0.75 IU/L. However, no significant differences were found.

**Table 3:**
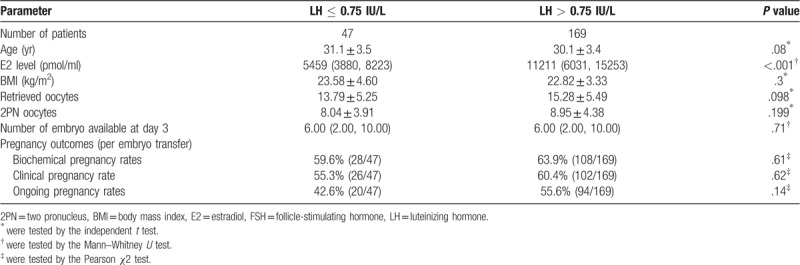
Comparison of pregnancy outcomes in the group 1 (FSH-only group) between patients with LH level lower than 0.75 IU/L versus those with LH level higher than 0.75 IU/L.

Table 4 showed the pregnancy rates among the 3 groups in patients whose residual endogenous LH levels after down-regulation were lower than 0.75 mIU/ml. There were 47, 42, and 23 patients who received embryo transfer and whose residual endogenous LH levels after down-regulation were lower than 0.75 mIU/ml in Group 1, 2, and 3, respectively. The biochemical, clinical and ongoing pregnancy rates in Group 3 (FSH+rLH) were 60.9%, 60.9%, and 47.8%, respectively, all of which were a little higher than those in Group 1 and Group 2. However, there were not any significant differences.

**Table 4:**
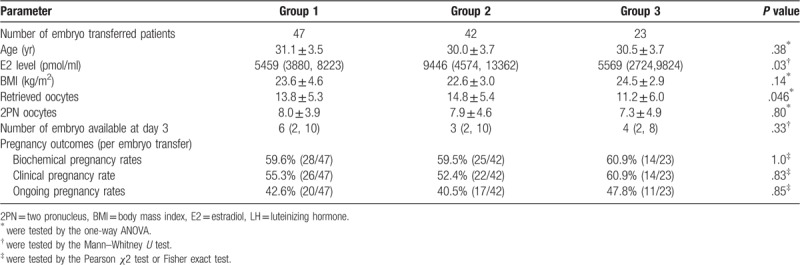
Comparison of pregnancy outcomes in patients with endogenous LH less than 0.75 IU/L in the 3 groups.

## 4 Discussion

The issue regarding LH supplementation in assisted reproductive techniques (ART) cycles has been a matter of debate for years. Currently, there are mainly 2 types of LH, rLH, and HMG. Which type of LH should be added and when should the supplementation of LH be started? There are still controversies about these questions. ^[17]^ In the present study we compared the effect of the rLH and HMG supplementation at an early time of follicular development on IVF outcomes in a GnRH agonist protocol among women of normal ovarian function undergoing their first IVF/ICSI cycle. Our study showed no significant increase in biochemical, clinical and ongoing pregnancy rates after addition of either HMG or recombinant LH. This was generally consistent with the systematic review and meta-analysis reported by Kolibianakis.^[14]^

The favorable effect of LH addition in combination with FSH for follicular stimulation during IVF in poor responders or older women has been supported by some studies.^[17–19]^ Alberto Revelli's group ^[4]^ also reported that the addition of LH at early and mid-follicular stage have comparable effects on the IVF outcome in poor responders. However, in normogonadotropic women, the effect of LH addition remains unsettled. A systematic review performed to evaluate whether endogenous LH levels predicted the likelihood of ongoing pregnancy beyond 12 weeks in women undergoing ovarian stimulation for IVF found that low endogenous LH levels were not associated with a significantly decreased probability of ongoing pregnancy.^[14]^ This systematic review and meta-analysis is generally consistent with our results.

It was reported that LH could induce the theca cells to enhance the androgen and polypeptide growth factor production on the developing follicles which leads to promotion of follicular responsiveness to FSH during follicular recruitment and selection.^[4,5]^ However, in this study, the supplementation of either rLH or HMG did not increase the ovarian response including the total days of gonadotropin treatment, E2 level on HCG administration day. This could be explained by the fact that residual endogenous LH levels after down-regulation might vary from each other.

Additionally, some studies chose 0.5 IU/L as the LH threshold and found lower LH level was associated with lower implantation and pregnancy rates. ^[11,20]^ However, in the present study, the number of patients whose LH level were lower than 0.5 IU/L was too limited to be analyzed to make a reasonable conclusion. Since there was not acknowledged standard for the LH threshold and different hospitals had different LH detection results, we made the quartile of LH value of 0.75 IU/L as the threshold, based on the LH distribution of our study, to investigate whether LH level was associated with the IVF outcomes and whether LH addition was conducible to improve the IVF outcomes in patients whose LH levels were relatively low. Our results showed the biochemical, clinical and ongoing pregnancy rates were not associated with residual endogenous LH levels. In addition, LH supplementation did not significantly improve the IVF outcomes. The inconsistent results between this study and previous studies might be ascribed to the different LH threshold set and different LH measurement.

Although both 2 forms of LH addition did not exhibit any superiorities in IVF outcomes compared to rFSH alone, it may be cost-effective for the clinicians to take the advantage of HMG addition instead of rFSH alone in normal ovarian reserve patients as an alternative choice since HMG is much cheaper than rFSH. However, there were still some potential limitation in the present study, which could not be neglected. First, the nature of recombinant LH, HMG, and recombinant FSH administration in separate injections and the need for the physician to adjust dosing based on the different response to medication may influence the final results. Secondly, owing to the specific retrospective study design, bias cannot be ruled out. Because of the limitation mentioned above, the clinical significance of this result is a matter of judgment for clinicians in practice. In the future, multicenter randomized prospective study and larger sample size are required in order to confirm the result.

## 5 Conclusion

In conclusion, our retrospective study showed that either rLH or HMG supplementation at early follicular phase produced no significant benefit in normogonadotropic women undergoing their first cycle of IVF/ICSI.

## Author contributions

**Conceptualization:** Xi Xia.

**Formal analysis:** Xi Xia, Liyuan Tao.

**Funding acquisition:** Hongzhen Li.

**Investigation:** Yu Shi, Lan Geng, Zhenhui Hou, Haiyan Wang.

**Methodology:** Dan Liu, Weiping Qian.

**Project administration:** Hongbo Lin, Fanhua Meng, Weiping Qian.

**Supervision:** Lan Geng, Jian Da, Jie Qiao, Hongzhen Li.

**Validation:** Lan Geng.

**Writing – original draft:** Xi Xia.

**Writing – review & editing:** Rong Li, Yun Chen, Hongzhen Li.
